# *In vivo* microscopy as an adjunctive tool to guide detection, diagnosis, and treatment

**DOI:** 10.1117/1.JBO.27.4.040601

**Published:** 2022-04-27

**Authors:** Kevin W. Bishop, Kristen C. Maitland, Milind Rajadhyaksha, Jonathan T. C. Liu

**Affiliations:** a University of Washington, Department of Bioengineering, Seattle, Washington, United States; b University of Washington, Department of Mechanical Engineering, Seattle, Washington, United States; c Texas A&M University, Department of Biomedical Engineering, College Station, Texas, United States; d Memorial Sloan Kettering Cancer Center, Dermatology Service, New York, New York, United States; e University of Washington, Department of Laboratory Medicine and Pathology, Seattle, Washington, United States

**Keywords:** *in vivo* microscopy, clinical translation, commercialization, multimodal imaging, artificial intelligence

## Abstract

**Significance:**

There have been numerous academic and commercial efforts to develop high-resolution *in vivo* microscopes for a variety of clinical use cases, including early disease detection and surgical guidance. While many high-profile studies, commercialized products, and publications have resulted from these efforts, mainstream clinical adoption has been relatively slow other than for a few clinical applications (e.g., dermatology).

**Aim:**

Here, our goals are threefold: (1) to introduce and motivate the need for *in vivo* microscopy (IVM) as an adjunctive tool for clinical detection, diagnosis, and treatment, (2) to discuss the key translational challenges facing the field, and (3) to propose best practices and recommendations to facilitate clinical adoption.

**Approach:**

We will provide concrete examples from various clinical domains, such as dermatology, oral/gastrointestinal oncology, and neurosurgery, to reinforce our observations and recommendations.

**Results:**

While the incremental improvement and optimization of IVM technologies should and will continue to occur, future translational efforts would benefit from the following: (1) integrating clinical and industry partners upfront to define and maintain a compelling value proposition, (2) identifying multimodal/multiscale imaging workflows, which are necessary for success in most clinical scenarios, and (3) developing effective artificial intelligence tools for clinical decision support, tempered by a realization that complete adoption of such tools will be slow.

**Conclusions:**

The convergence of imaging modalities, academic-industry-clinician partnerships, and new computational capabilities has the potential to catalyze rapid progress and adoption of IVM in the next few decades.

## 1 Introduction and Motivation

In the early days, the field of *in vivo* optical microscopy (or “optical biopsy”) was optimistically touted as a potential replacement for conventional *ex vivo* histology that could be noninvasively and rapidly performed at the point of care (e.g., outpatient clinic) or point of procedure (e.g., operating room). After several decades of research progress, this original vision has been refined into a more nuanced relationship between *in vivo* microscopy (IVM) and standard histopathology. IVM is now being seen as a complementary method to evaluate tissue morphology, along with dynamic processes such as blood flow, leukocyte trafficking, oxygenation, and metabolism, rather than as a wholesale replacement for histology. This is based on a realization that the strengths of both *ex vivo* histology and IVM can be leveraged synergistically to improve patient care. Concurrently, the practice of *ex vivo* histology is also being transformed with rapid technological advances, including non-destructive three-dimensional (3D) assessments of large tissue volumes (e.g., entire biopsies),^1^^–^^3^ spectral imaging techniques,^4^^–^^6^ multiplexed molecular analyses (e.g., spatially resolved genomics and transcriptomics),^7^^–^^9^ and rapid assessments of fresh tissue specimens (e.g., structured illumination microscopy and microscopy with ultraviolet surface excitation).^10^^–^^12^

A number of key challenges associated with conventional histology present opportunities for IVM to serve as a valuable adjunct. First, for the early detection of malignancies, such as oral cancer and skin cancer, a brute-force strategy is to “physically sample” all visually suspicious lesions via biopsy and histology. However, the majority of such suspicious lesions are benign,^13^^,^^14^ which makes invasive biopsy challenging to justify when considering time/cost considerations and patient risk. Therefore, malignant lesions (e.g., oral lesions) may remain either undetected or inadequately managed until they progress to a more-advanced and less-treatable stage. This is especially true in resource-limited settings and is a classic example of trying to find the optimal balance between minimizing mortality/morbidity and also trying to manage healthcare costs and quality of life for patients. A related issue is that the histological examination of biopsies and surgical specimens frequently suffers from limited “microscopic sampling” of the tissue specimens, which can make early detection, diagnosis, and grading difficult for spatially heterogeneous diseases. For example, if only a few 5-μm sections are viewed from a 1-mm diameter needle biopsy, this results in <1% of the tissue volume being analyzed. In light of these shortcomings of conventional biopsy and histopathology, real-time IVM presents an opportunity to efficiently sample many regions of tissue noninvasively, and to screen for lesions that are potentially malignant to guide subsequent biopsy and gold-standard pathological analysis (i.e., image-guided biopsy).

Another challenge associated with conventional histology is the long turnaround time between specimen collection and results (typically days for permanent sections and >30  min for frozen sections). This is especially problematic for surgical applications, where frozen section analysis (FSA) is not always preferred or accurate compared to post-operative pathology. For example, most breast cancer resections rely upon postoperative pathology—formalin fixed and paraffin embedded sections—to confirm that negative surgical margins are achieved. This is due to the inferior image quality of intraoperative frozen sections for lipid-rich breast tissues. Studies show that >20% of breast cancer lumpectomy patients must return for re-excision surgeries due to positive margins,^15^^,^^16^ which is costly and risky/traumatic for patients. While FSA is within the standard of care for intraoperative guidance of many tissue types, problems include: (1) poor image quality, (2) slow turn-around times when the frozen section lab is not integrated with the surgical suite, and (3) loss of specimen orientation (with respect to the surgical wound) due to transport of the specimen to a remote lab. IVM has the potential to provide preliminary results in real time to guide surgery, especially for applications in which precision is paramount (e.g., neurosurgery, resections at cosmetically sensitive locations).

In summary, it is clear that IVM does not need to entirely replace conventional histology to add clinical value. Rather, IVM can provide non-invasive imaging data that can be appropriate to guide specific interventions, to provide a preliminary diagnosis, or to serve as a screening-and-triage tool whose results can later be confirmed with gold-standard histology. We draw our examples here from purely optical techniques that can serve in this complementary role, with a specific focus on imaging methods that offer cellular-level to subcellular-level resolution and depth-resolved imaging (i.e., optical sectioning). Though we do not address them in detail, there are many other promising *in vivo* imaging methods that are actively being investigated, including photoacoustic imaging,^17^^–^^19^ optical coherence tomography (OCT),^20^ and a variety of non-depth-resolved wide-field-microscopy approaches.^21^^,^^22^

Examples of IVM technologies that we focus on in this article include confocal and nonlinear microscopes in an endoscopic, handheld, or gantry-mounted configuration, deployable at the point of care or point of procedure for early disease detection (screening) and therapy guidance. In some cases, the goal of IVM may be to deliver images that are similar to conventional histology for early triage of suspicious lesions and for surgical guidance. In other cases, the goal may be to quantify sparse signatures from endogenous and exogenous contrast agents in a way that is not possible with conventional wide-field (low resolution) surgical microscopes, e.g., sub-cellular protoporphyrin IX (PpIX) expression patterns at diffuse glioma margins.^23^^,^^24^

Whereas biopsy and pathology provide a static snapshot of a disease, IVM is capable of detecting and monitoring dynamic changes noninvasively at various time points (anywhere from seconds to months apart). This capability clearly distinguishes IVM from *ex vivo* histopathology and can provide significant “added value” for diagnostic utility. However, to date, the capacity to image dynamic changes remains under-developed and under-utilized. Limited examples include the dynamic imaging of blood flow and cellular metabolism, which can reveal subtle physiological and pathological features,^25^^,^^26^ as well as real-time visualization of dynamic cellular responses to therapeutic interventions.^27^ Similarly, longitudinal examination of patients with IVM can be useful for the surveillance of suspicious lesions. Pediatric populations, for example, exhibit frequent evolution, progression and/or regression of features such as skin moles, especially during their adolescent years.^28^^–^^30^ Various forms of longitudinal imaging (other than IVM) and periodic biopsy/cytology are already performed in many clinical settings such as prostate (e.g., active surveillance for indolent prostate cancers), cervical (e.g., screening via Pap smear), and gastrointestinal care (e.g., monitoring in Barrett’s esophagus),^31^^–^^33^ both for screening and for monitoring for progression to dysplasia and malignancy. Noninvasive IVM technologies have an obvious role in such settings to reduce biopsy and over-diagnosis by triaging only high-risk sites for invasive biopsy and histopathology. Furthermore, longitudinal IVM may be used to monitor response to various therapies without biopsy, such as endoscopic mucosal or radiofrequency (RF) ablation, photodynamic therapy, skin pigmentation disorder treatments, and others.^34^^–^^37^

**Fig. 1:**
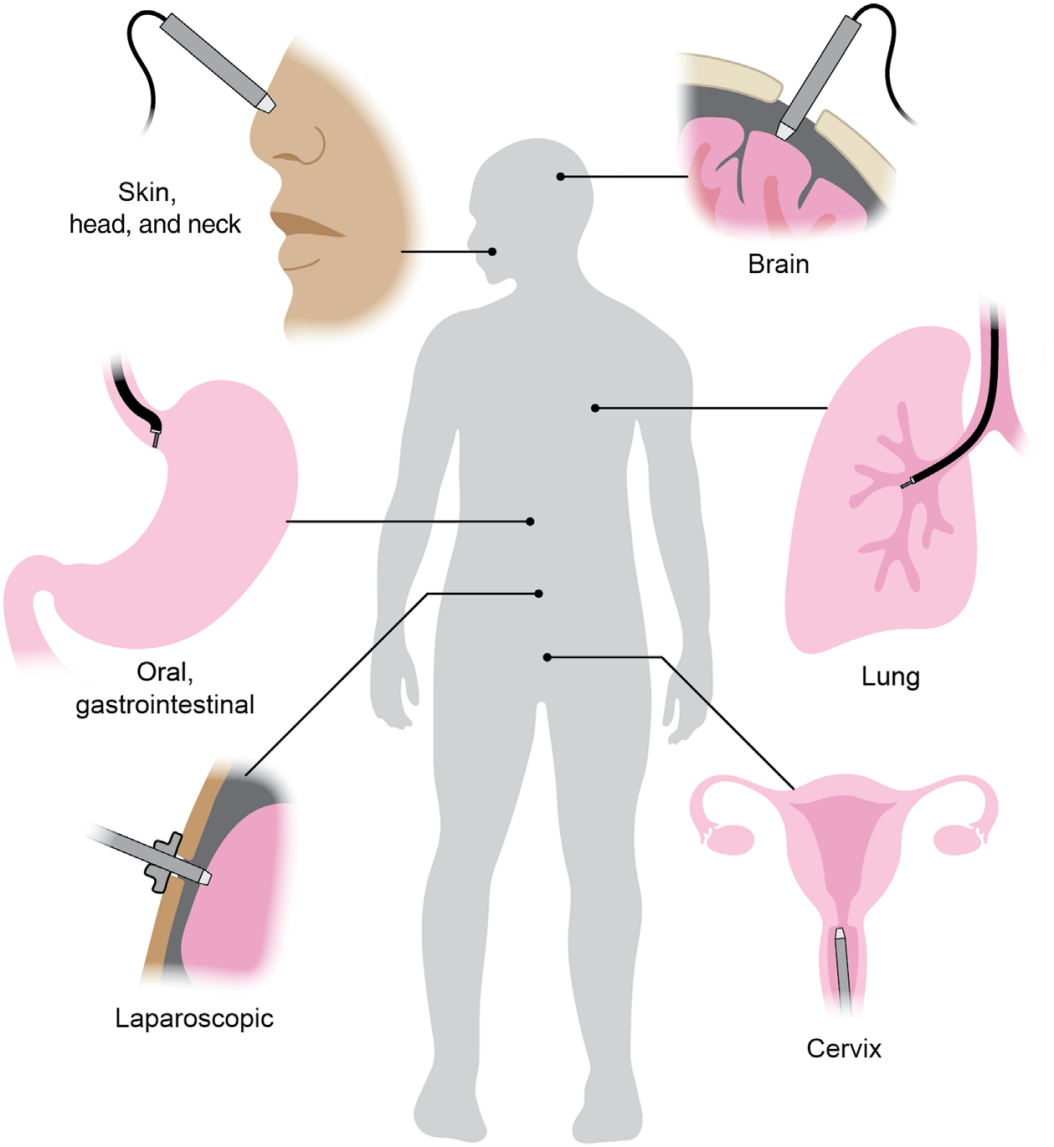
Various clinical applications of IVM. IVM devices can be handheld, manipulated robotically, or deployed through a flexible endoscope or rigid laparoscopic device. Figure created by Jacki Whisenant.

Rather than providing a technical or historical review of IVM technologies and clinical applications, we offer a perspective on key challenges and opportunities for clinical translation. While there are many clinical use cases of IVM (Fig. 1), we will mainly follow three illustrative applications (dermatology, oral/gastrointestinal oncology, and neurosurgery), for which major efforts have been made to translate IVM techniques into routine clinical practice. For technical background, we direct readers to excellent recent reviews on IVM.^21^^,^^23^^,^^38^^–^^41^

## 2 Translational Challenges

### 2.1 Technical Considerations for *In Vivo* Microscopy

**Fig. 2:**
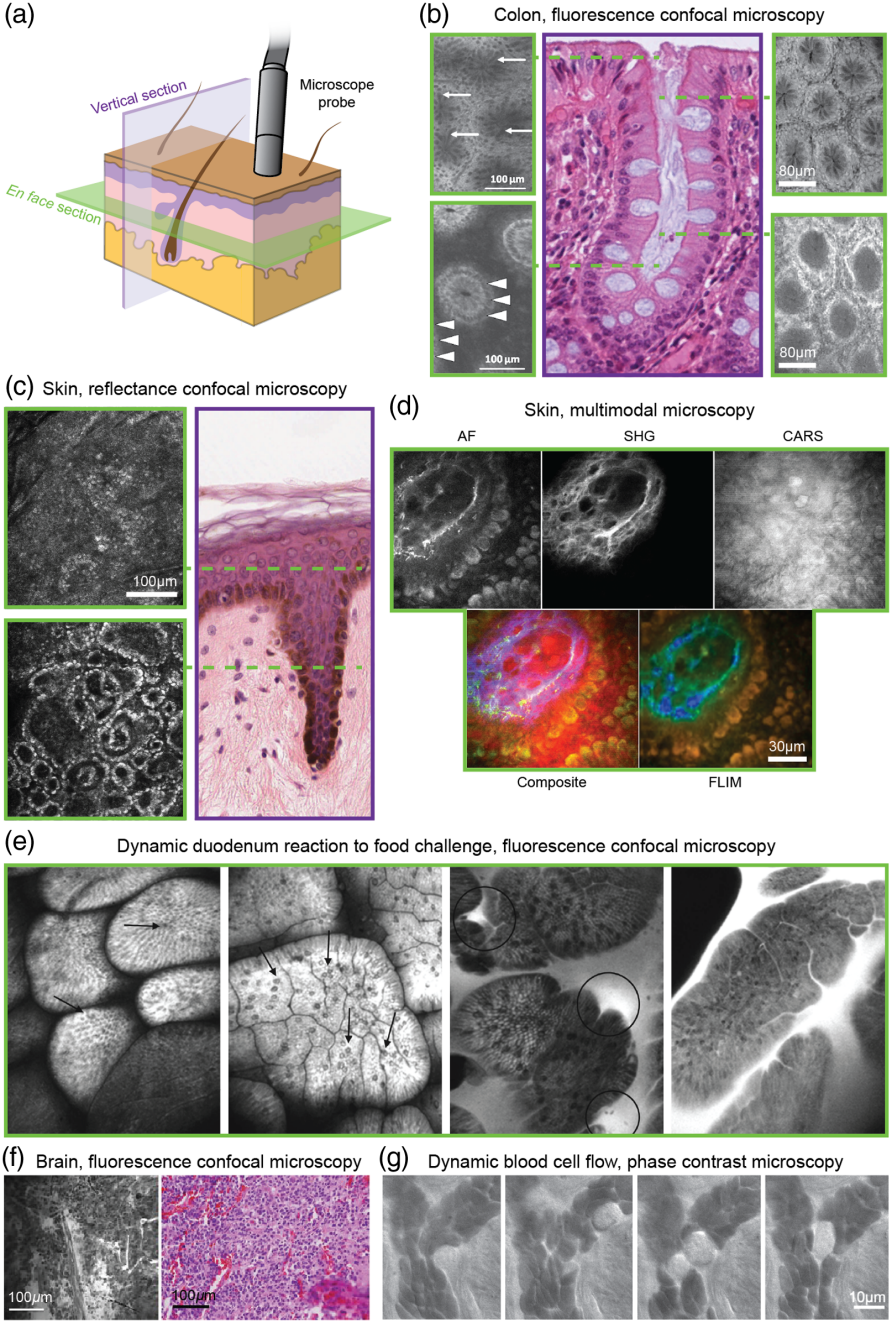
Atlas of IVM images. (a) In contrast to conventional histology, in which vertical tissue sections (purple) are most common, most IVM techniques provide *en face* tissue images (green). Figure created by Jacki Whisenant. (b) *En face* images of colonic crypts at various depths using endoscopic fluorescence confocal microscopy. Annotations indicate dark mucin (top left, arrows) and basal cell nuclei (bottom left, arrowheads). A representative vertical histology section in which the dashed lines indicate the approximate imaging depth of various *en face* IVM images. Left images were collected using topical cresyl violet dye, while right images used intravenous fluorescein sodium. Left: reprinted from Ref. 49 with permission from the American Society for Gastrointestinal Endoscopy. Center: reprinted with permission from Ref. 50; Copyright Regents of the University of Michigan: CC BY-NC-SA 3.0. Right: reprinted from 51 with permission from the American Gastroenterological Association. (c) *En face* images of skin using RCM showing the epidermis (top) and dermal–epidermal junction (bottom). A representative vertical histology section indicates approximate imaging depth. Left image provided by Dr. Kivanc Kose and Dr. Aditi Sahu, Dermatology Service, Department of Medicine, MSKCC. Right image reprinted with permission from Ref. 52; Copyright Regents of the University of Michigan: CC BY-NC-SA 3.0. (d) *En face* images of skin (dermal papillae) using multimodal microscopy reveal various morphological features via AF, SHG, CARS, and FLIM colored by mean lifetime. Reprinted from Ref. 53. (e) *En face* images of duodenum via fluorescence confocal microscopy in a patient with irritable bowel syndrome (IBS) allow dynamic visualization of response to a food challenge. A healthy control at baseline (left) shows low overall levels of intraepithelial lymphocytes (arrows) compared to a patient with IBS at baseline (center left). Following food reaction, the patient with IBS shows leakage of fluorescein-labeled plasma (circles) and increased lymphocyte numbers (center right, right). Reprinted from Ref. 27 with permission from the American Gastroenterological Association Institute. (f) Intraoperative fluorescence confocal microscopy of anaplastic oligodendroglioma following intravenous administration of fluorescein sodium with corresponding histology of the same imaging site. Reprinted from Ref. 54, figure used with permission of Journal of Neurosurgery Publishing Group, Copyright-protect and excluded from CC BY 4.0 licensure. (g) Time-course images of a tongue capillary using oblique back-illumination capillaroscopy (phase contrast) showing the dynamics of red and white blood cells. Reprinted from Ref. 25. All IVM images shown are collected *in vivo* in humans.

Most IVM devices generate images in the *en face* orientation, or parallel to the surface of the tissue. While these *en face* images are orthogonal to standard histology sections (typically vertical cross sections), the use of axial scanning,^42^^–^^45^ oblique orientation of the imaging plane,^46^ spectral encoding of depth,^47^ multiplane scanning,^48^ or other methods to adjust imaging depth can enable effective evaluation of cytomorphology and cellular arrangement through the desired layers and volume of tissue. A number of examples are shown in Fig. 2. Given that pathology traditionally relies on examination of vertically oriented sections, the utility of *en face* images was, not surprisingly, questioned and thought to be a limitation in early years. However, as initial clinical studies progressed into larger studies and trials, the value of examining morphology through *en face* images and mosaics became more evident. For example, in the context of skin cancer, melanomas and other types of lesions (lentigo maligna, Paget’s disease) often present with substantial focal areas and lateral spread. The ability to quickly survey a range of lateral fields of view with IVM, along with the acquisition of image stacks (as a function of depth), is highly valuable for characterizing these types of lesions and enables the sampling of tissue volumes that are 5× to 10× larger than what is achieved with conventional biopsies and slide-based histopathology, all within minutes at the patient bedside.^14^ This volumetric sampling approach is now being routinely implemented for image-guided diagnosis of skin lesions: i.e., the acquisition of 3 to 5 *en face* images along with 3 to 5 depth stacks, where clinical trials have shown that detection specificity improves from 50% to 70%.^14^

For the IVM techniques being considered in this perspective, spatial resolution is on the order of ∼1  μm in the lateral direction and ∼5  μm in the axial direction (depth direction) into the tissue. These dimensions enable real-time visualization of cellular features and tissue architecture. In particular, nuclear morphology is critical for the diagnosis of many pathologies. With the high lateral resolution mandated for pathology-level examination, the native field of view of a portable IVM device is typically limited to $<1\;{\mathrm{mm}}^{2}$, which is small relative to the large ($∼{\mathrm{cm}}^{2}$) tissue areas of interest. Nonetheless, rapid imaging of small fields of view can enable many tissue locations to be noninvasively interrogated, providing real-time information about suspicious tissues to guide biopsy-site selection,^55^ such as for oral cancer screening, as previously mentioned.^56^

**Fig. 3:**
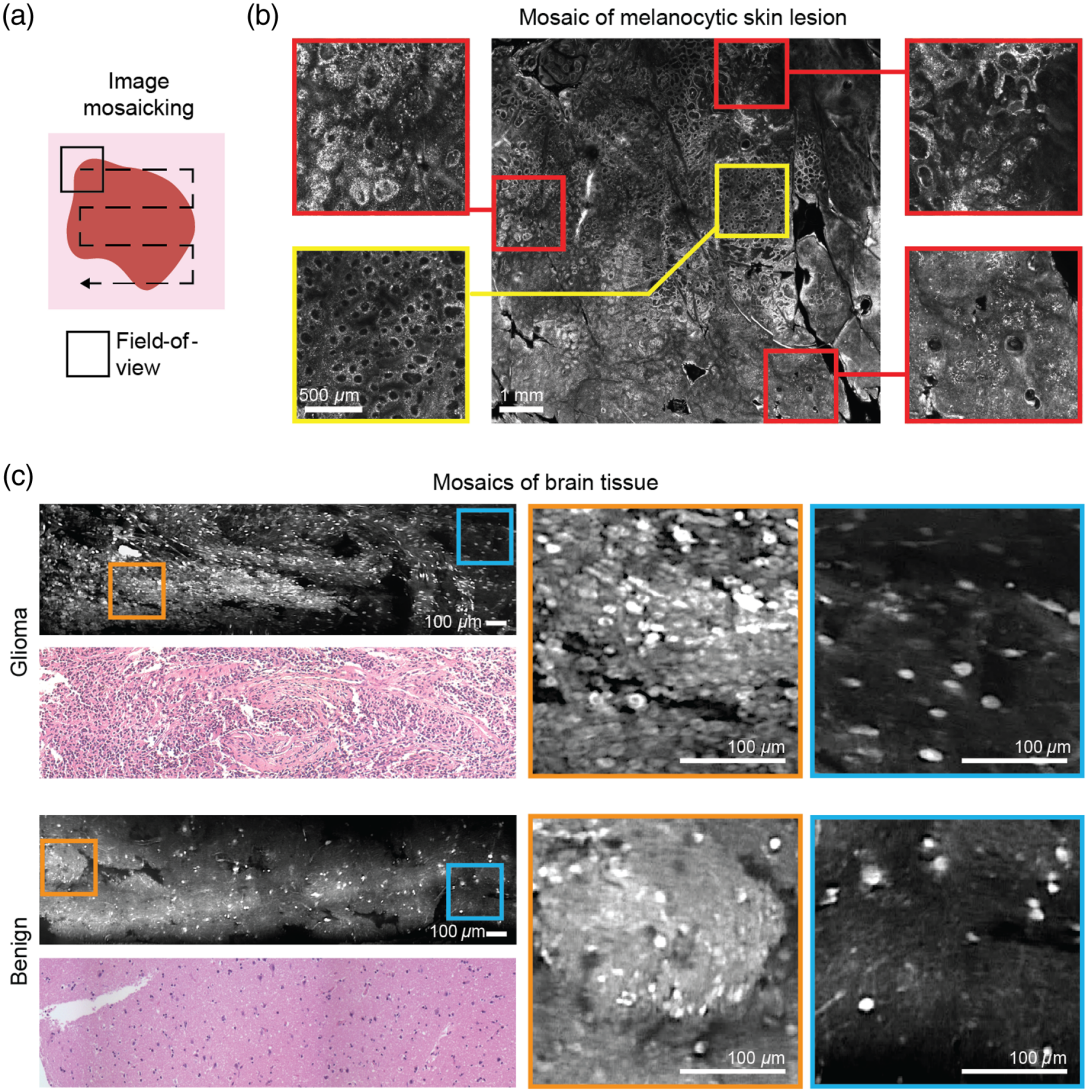
(a) Image mosaicking combines small, high-resolution fields of view (equivalent to 20× to 40× magnification using a typical pathology microscope) to provide larger effective fields of view (equivalent to 2× to 4× magnification). (b) *In vivo* RCM mosaic displaying 8  mm×8  mm area (equivalent to ∼2× magnification) of a heterogeneous melanocytic lesion, acquired at the bedside in ∼50  s. Red-boxed zoomed-in regions show focal areas of malignancy/cancer compared to the yellow-boxed regions showing the normal ring-like patterns of basal cells in the epidermis. Mosaic provided by Drs. Aditi Sahu, Yuna Oh, Miguel Cordova, and Veronica Rotemberg, Dermatology Service, Department of Medicine, MSKCC. (c) Fluorescence confocal microscopy mosaics of *ex vivo* brain tissue from a glioma patient stained with acridine orange. While individual fields of view may show similar features between glioma (top) and benign (bottom) tissues, large mosaics show clear differences in overall cytoarchitecture between the two specimens. Adapted from Ref. 59; CC BY 4.0.

Limitations in field of view have been mitigated by sophisticated mosaicking techniques,^46^^,^^57^^–^^59^ typically at the tissue surface or at a fixed subsurface depth [Fig. 3(a)]. *En face* mosaics allow clinicians to assess tissue in a hierarchical lower-to-higher magnification workflow as is routine in conventional *ex vivo* pathology, using overall tissue architecture and gross patterns of cellular and nuclear morphology to first identify regions of interest for subsequent higher-resolution examination of cellular and nuclear detail [Figs. 3(b) and 3(c)]. This is especially useful for examining heterogeneous tissues, such as focal areas of malignancy that often spread laterally, or the lateral margins of a tumor. Large-area mosaics also play a role in procedure guidance, enabling highly accurate and repeatable biopsy sampling of focal malignant areas for traditional and molecular pathology (genomics, ribonucleic acid-sequencing, and flow cytometry).^60^^–^^62^ A challenge to *in vivo* mosaicking is the need to minimize motion artifacts and abrupt changes in imaging position. These issues can be mitigated somewhat by increasing the frame rate of imaging (to reduce motion blur)^63^ and by stabilizing the imaging probe, for instance with robotic assistance.^64^^,^^65^

Subcellular spatial resolution also limits imaging depth to at most a few hundred microns for most *in vivo* microscopes, beyond which image quality is substantially degraded by tissue scattering and aberrations. While this is sufficient to image epithelial tissues (both externally and internally via endoscopy/surgery), where many cancers occur, a variety of clinical scenarios (e.g., determining the extent of tumor invasion) would benefit from increased imaging depth. Strategies to increase usable imaging depth, such as with multimodal imaging approaches (see Sec. 3.1),^66^^–^^68^ have the potential to expand the clinical applications of IVM.

In summary, while the advantages of IVM include the ability to resolve subcellular features of tissue *in vivo*, the imaging is often limited in field of view and depth of imaging. There are inherent trade-offs between spatial resolution and field of view, device size, and imaging speed. Devices are typically designed to be application-specific due to these limitations and trade-offs. Depending on the organ or tissue of interest and specific clinical application, design specifications, such as spatial resolution in two dimensions or 3D, field of view, imaging depth, imaging speed, and device size must be carefully optimized. Technical advances to mitigate and balance these trade-offs for particular applications may therefore be as important as incremental improvements in any single design specification. In addition to these technical design challenges, device cost and clinical usability also factor into the successful clinical adoption of these devices (as described in later sections).

### 2.2 Endogenous and Exogenous Contrast Mechanisms

Compared to the diverse library of stains available for *ex vivo* pathology, IVM at present is restricted to either endogenous sources of contrast or a small number of exogenous contrast agents. Endogenous sources of contrast, such as backscatter (“reflectance”) and autofluorescence (AF), are attractive as they enable IVM without having to introduce stains or contrast agents into humans, which present additional toxicity, regulatory, and logistical challenges. For example, reflectance confocal microscopy (RCM), OCT, and optical coherence microscopy (OCM) imaging are based on the detection of primarily singly back-scattered photons with contrast produced by refractive-index variations in the stroma, cells, and subcellular organelles.^69^^,^^70^ In skin, highly scattering cellular cytoplasm (keratin, melanin, and organelles) appears as regions of strong, relatively uniform and bright signal, whereas nuclear chromatin and nucleoli appear as regions of highly variable (by up to ∼4 orders of magnitude) dark signal. Some tissue features, including certain cell types, are difficult to differentiate based on reflectance contrast alone, imposing a fundamental limit on diagnostic accuracy, and especially specificity. As an illustrative example, a large number of clinical studies exploring reflectance imaging for detection of basal cell carcinomas have revealed that the detection specificity of RCM and OCT ranges from 50% to 70%,^14^^,^^71^ a limit that might only be overcome with newer sources of contrast. AF is another endogenous source of contrast and has been popular with multiphoton microscopy.^72^^,^^73^ However, AF is weaker than backscattered light, and can impose limits on imaging speed and field of view.

Some of the limitations of endogenous contrast may be overcome with the use of exogenous contrast agents, including molecularly targeted agents.^74^ An exogenous contrast agent to be used *in vivo* should ideally provide high cellular or nuclear specificity, exhibit low toxicity, and be detectable from within strongly scattering tissue when imaged at high speed with a high-resolution microscope. Optical contrast agents that are approved for human use by the Food and Drug Administration (FDA) are limited in number and are primarily nonspecific. After decades of use in chromoendoscopy to visualize tissue surface features based on dye absorption, methylene blue has more recently been applied as a near-infrared (NIR) fluorescence contrast agent in surgical microscopy and IVM.^75^ Intravenous fluorescein permits visualization of vasculature and tissue architecture.^51^^,^^76^^–^^78^ Indocyanine green, a nonspecific NIR fluorescent dye used intravenously for angiography, has had great success in surgical guidance and intraoperative decision making through advances in NIR optical imaging in surgical microscopes and laparoscopes.^79^ While these nonspecific fluorescent dyes are clinically used with wide-field (low resolution) endoscopes and surgical microscopes, targeted molecular imaging agents may be better at leveraging the cellular-resolution capabilities of IVM as they can reveal characteristics (morphology, prevalence, etc.) of specific cell populations of interest. There are a large number of clinical trials to evaluate targeted contrast agents that incorporate FDA-approved dyes or similar fluorophores with various targeting ligands.^74^ For example, for intraoperative use to guide the surgical resection of gliomas, the FDA recently approved the use of 5-aminolevulinic acid (5-ALA), which induces preferential accumulation of a fluorescent metabolite, PpIX, in glioma cells.^80^^,^^81^ Recent studies show that IVM tools have the sensitivity and resolution to detect the subcellular expressions of PpIX even in cases where wide-field surgical fluorescence microscopy fails.^24^^,^^82^

A high-contrast label that targets tumor nuclei could be highly attractive for surgical-guidance applications of IVM, allowing for both molecularly specific delineation of tumor cells as well as visualization of altered nuclear morphology (one of the hallmarks of cancer pathology). One example of a high-contrast nuclear-specific label is poly(adenosine diphosphate-ribose)polymerase inhibitor-fluorescent (PARPi-FL), a small molecule PARPi that is conjugated with boron-dipyrromethene fluorescent dye.^83^^,^^84^ PARPi-FL is a fluorescent nuclear marker, specific for the PARP1 DNA repair enzymes that are differentially overexpressed in oral pre-cancers and cancers. PARPi-FL has been shown to be non-toxic, was granted an investigational new drug status, and is currently being tested for the imaging of oral lesions.^85^

In summary, the development and refinement of exogenous targeted optical-imaging agents will likely play a role in the clinical translation of certain IVM tools in the future. While this provides an attractive opportunity to advance the field of IVM, there are also complexities with regards to the regulatory approval of device/agent combinations and technical challenges to overcome. The signal (i.e., number of photons) available for detection from any contrast agent is intrinsically impacted by factors such as the relative sparsity of structures labeled by targeted agents (e.g., nuclear chromatin and proteins, cell surface receptors, or cellular organelles) and scattering losses in thick tissues. Overcoming such intrinsic factors requires jointly optimizing both contrast agents and IVM imaging devices.

### 2.3 Clinical and Institutional Considerations

Early engagement and long-lasting high-quality collaboration between researchers, industry experts, and “early adopter” clinicians is critical for translational success. A poor understanding of the needs, realities, and psychology of clinical practitioners can often hamstring the adoption of a powerful new technology. One example is Mohs micrographic surgery for the removal of non-melanoma skin cancers. There have been efforts in academia and industry to develop IVM technologies, as well as rapid *ex vivo* microscopy techniques, to either serve as an adjunct or to replace FSA, which is the current standard-of-care technique to guide Mohs procedures.^86^^–^^89^ At first glance, a faster and simpler alternative to FSA seems like an appealing way to streamline Mohs surgery. In the United States, however, Mohs surgery is already highly efficient, with operating rooms and FSA labs that are adjacent and integrated. Parallel Mohs procedures occur in adjacent operating rooms, and surgeons are often trained to read histology slides on the spot. Combined with lucrative reimbursement rates for Mohs procedures, there is little incentive to modify the existing FSA workflow. This is not the case in much of Europe, where the pathology lab is often offsite and the frozen sections must be read by an independent pathologist, such that the FSA process can take 30 to 60 min. This incentivizes adoption of novel IVM approaches as illustrated by the large number of publications on IVM for Mohs surgery from our dermatologist colleagues in Europe.^88^^,^^89^ After this success, confocal microscopy developed for Mohs surgery is now being applied in other settings such as urologic surgery (prostate biopsies and excisions) and breast surgery (core needle biopsies).^10^^,^^90^^–^^94^ Frozen sections cannot be readily prepared and/or may not be reliable in these settings, necessitating reliance on fixed sections (which take at least a day to prepare), thereby, presenting an even greater motivation for IVM and rapid *ex vivo* tissue microscopy technologies.

Institutional barriers and the mindset of clinicians must also be carefully assessed when trying to develop any new medical technology. As a broad generalization, certain fields of medicine are more embracing of new technologies and risk-tolerant than others. Many factors are involved here, including how mature the clinical field is, how cohesive the community is within that specialty, how well the existing infrastructure works, and how well-reimbursed existing practices are. For example, the field of breast cancer surgical oncology is relatively mature, as driven by nearly half a century of broad public awareness and the high incidence of breast cancer. However, being a mature field does not mean that there is a lack of controversy, as position papers are frequently being published on what constitutes an adequate surgical margin for various breast cancer subtypes (e.g., tumor on ink for invasive carcinoma versus >2-mm margins for ductal carcinoma *in situ*).^15^ Given the large number of stakeholders in the breast cancer field, including large numbers of patient advocacy groups and clinicians, it can be challenging for new technologies to be accepted into the standard of care. On the other hand, the neurosurgery field has been more embracing of new technologies, driven in part by the dire prognosis for patients with diffuse gliomas, with minimal improvements in patient outcomes in recent decades.^95^ The neurosurgeon community is also close-knit and relatively small compared to other oncology fields, allowing clinical adoption to be accelerated. An example is the recent FDA approval of fluorescence-guided surgery for high-grade gliomas with 5-ALA, as described previously. This adoption process started in Europe following a landmark phase-3 clinical trial published in 2005,^80^ and demonstrates the ability of new optical microscopy technologies to be commercialized and adopted into clinical practice within a decade or so (albeit with low-resolution microscopy in this example).

## 3 Opportunities and Best Practices

### 3.1 Multimodal and Multiscale Imaging Workflows

Each optical imaging modality offers fundamental capabilities and limitations. This presents exciting opportunities to combine technologies into multimodal approaches such that the capabilities of one modality may complement and overcome the limitations of another. Using skin imaging as an example, RCM can visualize epidermal morphology with cellular resolution but is relatively slow and limited to imaging the superficial papillary dermis (∼200-μm deep). On the other hand, OCT is faster and can image into the reticular dermis (∼1-mm deep) but with resolution typically limited to visualizing architectural morphology (i.e., layers in the epidermis and underlying dermis) but not individual cells. These complementary technologies have been combined into a single device with co-registered fields of view.^66^ This approach has proven useful for imaging skin cancer, where RCM guides diagnosis by detecting basal cell carcinomas (BCCs), while OCT guides treatment by assessing a detected lesion’s margins and depth of invasion. In terms of guiding treatment, the deeper-imaging capability of OCT can allow superficial BCCs to be triaged for treatment with non-surgical approaches (e.g., laser ablation) as opposed to the more-aggressive deeper subtypes that must be treated with Mohs surgery or traditional surgical excision.^67^^,^^96^ A combined RCM-OCT imaging approach is now proving capable of allowing integrated diagnosis-and-treatment of BCCs in a single patient visit, leading to a “one-stop shop” patient-care paradigm (image-guided therapy).^67^

Other multimodal approaches include combining OCT/OCM, for reflectance imaging of cellular-scale tissue morphology, with either multiphoton AF microscopy or fluorescence lifetime imaging (FLIM) of cellular metabolism.^68^^,^^97^^–^^101^ OCT has also been combined with photoacoustic tomography for co-registered imaging of tissue morphology and vascular morphology.^102^ Likewise, nonlinear microscopy approaches [including two-photon AF, second-harmonic generation (SHG), and coherent anti-Stokes Raman scattering (CARS)] have been combined to visualize diverse tissue components in human skin.^53^

Combining imaging modalities may appear to be a straightforward task. However, in light of each modality’s fundamental limitations, designing and engineering devices for clinical applications poses non-trivial dilemmas. For quick and routine use on patients for rapid examination of large areas or volumes of tissue, maximizing the space-bandwidth product (total number of resolvable pixels) and frame rate of a microscope is necessary. However, optimal imaging parameters (e.g., frame rate and field of view) differ between modalities. For instance, the frame rates required for FLIM, or to image relatively weak AF signals in multiphoton microscopy, are necessarily lower than those required to image the relatively strong backscattered signals with RCM or OCT/OCM. Such trade-offs in imaging parameters must then be considered in the context of what minimum field of view, resolution, and sensitivity/specificity are needed to provide clinically actionable information.

Finally, various groups are working to co-register microscopic images with wide-area low-resolution clinical imaging modalities such as low-power surgical microscopy and magnetic resonance imaging (MRI). Here, the large disparity between spatial scales does not allow the images to be overlaid, but rather used in a hierarchical interpretive workflow. Most commonly, this would involve the rapid assessment of large tissue areas at low-resolution followed by the interrogation of localized regions of concern at high resolution. As an example, intraoperative neuronavigation is standard of care for brain tumor resections, in which a stereoscopic positioning system allows the position of surgical tools to be tracked and registered with preoperative MRI images in 3D. With neuronavigation, there is the ability to track the position of a handheld high-resolution microscope device that is placed in contact with the brain for real-time noninvasive histopathology, which could enable surgeons to integrate the best of whole-brain MRI and high-resolution microscopy to guide the resection of various brain lesions.^24^ In another example, for skin-cancer, a micro-camera for wide-field imaging has been integrated into the objective lens of an RCM device, providing a 4  mm×4  mm field of view to navigate the positioning of a confocal microscope with a field of view of 0.5  mm×0.5  mm.^103^

### 3.2 Academic-Industry Partnerships

Translating technologies into clinical settings can benefit greatly from partnerships with industry. While this may not be necessary for initial laboratory research to establish feasibility, industry partnerships become essential as technologies are scaled up to large, multicenter trials and are eventually commercialized (a requirement for wider dissemination and clinical adoption).^104^ Industrial partners are well-equipped to account for aspects central to commercializations, such as market size and dynamics, reimbursement/revenue models, regulatory approval, and manufacturing strategies. Deciding when to initiate an industry partnership requires balancing academic and commercial priorities. From an academic perspective, early industry input would be ideal. However, to mitigate initial risk, investors and other industrial stakeholders often prefer (or are mandated) to wait until early human studies are successful before engaging at a deep level. Navigating this “valley of death” is challenging, but a number of start-up ventures have been able to develop commercial products for early stage studies that have led to larger investments and well powered clinical trials. An early and now successful example is clinical RCM, where rapid progress from the first benchtop results, published in 1995,^105^ to the first commercialized clinical microscope [Vivascope, Caliber Imaging and Diagnostics (formerly, Lucid Inc.), Rochester, New York] in 1997^106^ supported the dissemination of the technology and initiation of large multicenter clinical studies and trials (Miami, Italy, Barcelona^14^).

One area where industrial involvement can be especially valuable is in refining the usability and ergonomics of a benchtop prototype for clinical testing and adoption. This usually means implementing the system as a handheld device, on a mobile cart, or in a gantry- or articulated arm-mounted configuration. Compared to a benchtop prototype, a clinical device must be robust and relatively easy to use while posing minimal disruption to established clinical workflows. While building a clinical device can and has been accomplished by academic groups, it can be useful to leverage industry expertise and resources to convert academic prototypes into more robust “product-like” devices (including technical support during clinical studies). A number of seemingly mundane details that are not usually considered by academic researchers, such as robust packaging, efficient cabling, user-friendly controls, sterilization, and set-up time/complexity, become critically important for clinical adoption.

Partnership with industry requires clear identification of an application and a clinical “pull” that complements the technological “push.” When developing a new device with limited resources, one must often focus on an initial application and “beachhead market.” However, an ideal scenario would be to transition the device into a “technology platform” that may be rapidly adapted and configured for diverse applications. Diverse applications, in turn, may require diverse sources of contrast, diverse approaches to interface with *in vivo* tissues, bespoke image-processing methods, and tailored clinical-workflow strategies, all of which require larger commercial infrastructures and investments.

For wide-scale acceptability and global adoption, cost often remains the single most difficult barrier to overcome. Cost, of course, scales down as acceptance, adoption, and use scale up, but therein lies the conundrum and risk for a new commercial device. This is one reason why medical devices are challenging for investors to include in their portfolios, as a rapid return on investment can be difficult to achieve. One common strategy for start-up ventures is to target preclinical and research markets, if available, as a source of initial revenue. While preclinical markets tend to be smaller, they allow for immediate and steady growth of a company while it continues to pursue clinical studies and adoption. An IVM-related example of this is the CellVizio confocal microendoscopy platform developed by Mauna Kea Technologies, which was initially marketed and sold to researchers for small-animal imaging prior to the dissemination and eventual regulatory approval of similar devices for a variety of clinical applications.

### 3.3 Computational Image Analysis

**Fig. 4:**
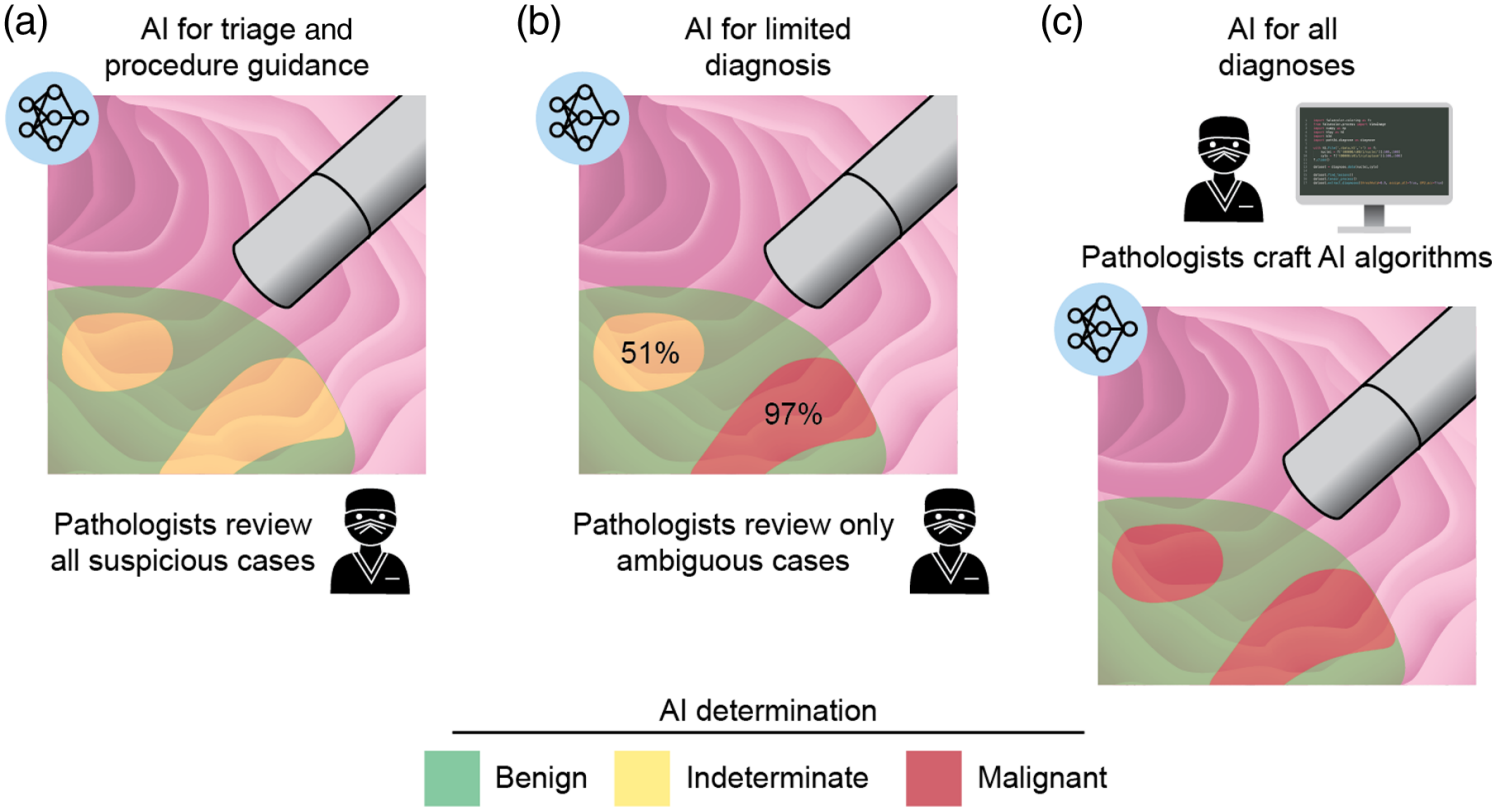
Stages of AI adoption in IVM in an endoscopic imaging example. (a) In the near term, AI will be used primarily for triage and procedure guidance, with all suspicious cases reviewed by clinicians. (b) As AI becomes more sophisticated, algorithms may label suspicious lesions based on the likelihood of malignancy such that pathologists need only review ambiguous cases. (c) Eventually, pathologists may be trained as data scientists and use their clinical expertise to craft and validate AI algorithms to automatically make the majority of diagnostic and prognostic determinations. Figure created by Jacki Whisenant.

No perspective on biomedical optical microscopy and imaging can be complete today without an appreciation for the current state and future possibilities of machine learning and artificial intelligence (AI) in general. Considering the capabilities of IVM devices to rapidly image and probe large volumes of tissue, AI approaches can help to manage the reading and analysis of increasingly massive amounts of imaging data. Initially, the obvious and likely most useful applications of AI will be for segmenting tissue structures and triaging images as benign/normal versus suspicious/malignant regions that should be carefully interpreted by a clinician [Fig. 4(a)]. Later stages could rely on AI methods for the automated diagnosis of simple unambiguous cases while flagging indeterminate/challenging cases for manual pathologist review [Fig. 4(b)]. A final stage of AI adoption, in which diagnostic and prognostic determinations are almost fully automated, is likely to occur first for niche applications, in which existing pathology approaches are not well developed, but could spread to larger and more-mainstream applications. For example, in radiology, FDA approvals are starting to be seen for AI-based screening of breast cancer and diabetic retinopathy.^107^^,^^108^ We and others anticipate that pathologists and radiologists of the future will increasingly be trained as data scientists who can leverage their clinical expertise to author and implement improved AI approaches in addition to validating them in their clinical practices [Fig. 4(c)]. Finally, it is worth noting that AI approaches may be particularly useful and popular in low-resource settings^109^ and could drive the wider adoption of IVM technologies in those parts of the world.

The performance of AI algorithms, and especially deep-learning approaches, depends on the completeness, quality, and generalizability of acquired training sets. In this respect, a significant challenge for the development of AI approaches will be the collection of large amounts of well-curated imaging datasets from diverse clinical settings. *In vivo* data acquired at the bedside is notoriously variable in appearance depending upon individual clinicians’ approaches, clinical workflows, and patient populations. Therefore, initial studies have often started with highly standardized datasets and well-defined patient sub-populations to simplify the development of accurate algorithms. As an example, deep-learning algorithms for analyzing dermoscopy images of skin lesions have been shown to perform as well as, and, in some studies, substantially better than dermatologists.^110^^–^^115^ Similar studies are being performed with RCM datasets of skin cancers.^116^^,^^117^ However, the initial success of AI in dermoscopy and in RCM for classifying skin lesions has relied on carefully selected training datasets, i.e., high-quality images from specialized academic centers of almost exclusively Caucasian skin.^114^^,^^118^ A major task for the foreseeable future will be to generalize these initial efforts and successes to reflect the reality of diverse clinical scenarios and patient populations. Key aspects to account for in dermatology applications will include different skin types and colors, rare skin conditions that do not appear in training datasets, and variations in image quality between settings. These variations in image quality could be due, for example, to differences in device settings (laser power, detector gain, etc.), user experience, patient-preparation protocols, and data-processing/storage methods. As a promising example to address these issues, a global academic-industry partnership effort, the International Skin Imaging Collaboration (ISIC), is working to establish a publicly accessible archive of skin images that is freely available. This project’s goal is to support development in AI as well as to promote standards for image acquisition, archiving, quality control, and interoperability (allowing use of images across diverse technologies and clinical platforms).^119^^,^^120^

Toward acquiring large datasets for rapid scalability and generalizability, AI efforts will most likely benefit from large-scale multi-site collaborations from the outset of a project, rather than the traditional approach of single-investigator-led efforts in individual laboratories and clinics. Such consortium efforts, when implemented on a truly large scale, could enable rapid harmonization and dissemination of AI methods. In parallel, imaging standards, akin to the digital imaging and communications in medicine (DICOM) standard in radiology, are needed to facilitate the development of device- and clinic-agnostic AI pipelines for IVM datasets. The ISIC approach serves as a laudable starting model in this regard that could be adopted for other IVM technologies and applications.

Advancing, fundamentally, from subjective and qualitative user-driven approaches to objective and quantitative standardized approaches at the bedside to guide data acquisition, image formation, and image annotation will be highly impactful for various imaging fields including IVM. In the immediate future, there are many applications of AI to improve the fundamental processes of image formation, especially for human interpretation. Given the inherent limitations of *in vivo* imaging in highly scattering tissues, advances in post-processing (e.g., denoising, artifact removal, contrast enhancement, and image translation) will be essential to supplement continuing improvements to raw image quality achieved through improvements in optical instrumentation. Emerging deep-learning-based methods such as Noise2Void^121^^,^^122^ and content-aware image restoration^123^ offer appealing strategies to enhance noisy IVM images without requiring changes to the instrumentation itself. Examples being explored for IVM specifically include denoising in images of skin lesions to facilitate high-speed multiphoton microscopy^73^ and RCM,^124^ as well as resolution enhancement of probe-based confocal laser endomicroscopy images.^125^ Likewise, recent work in *ex vivo* microscopy, such as deep-learning-based denoising of stimulated Raman scattering microscopy images^126^ and a similar approach to improve image quality in multiphoton microscopy images in signal-limited conditions,^127^ have the potential to translate to *in vivo* applications as well. Recent developments in image translation or style transfer, such as to convert label-free (e.g., AF) images to look like hematoxylin and eosin (H&E)-stained images,^128^ or to convert H&E-stained images to look like immunolabeled images,^3^ could also be valuable for intuitive interpretation by pathologists who are accustomed to certain tissue stains and appearances in histology images.

**Fig. 5:**
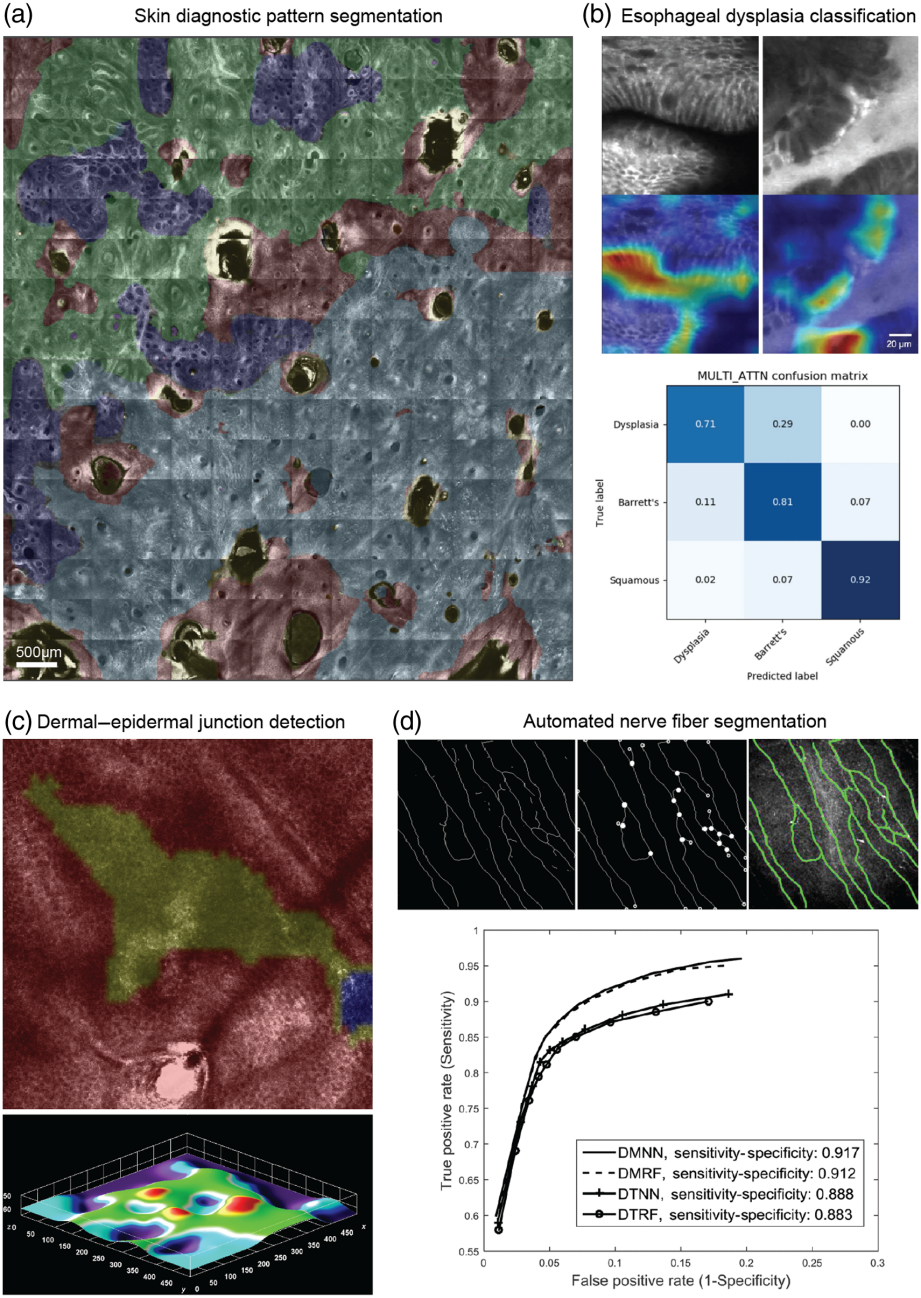
Many AI approaches in IVM to date have focused primarily on triage and procedure guidance rather than providing definitive diagnoses, though AI is increasingly being explored as a tool to augment human diagnostic/prognostic determinations. (a) Segmentation of diagnostic patterns in an RCM skin mosaic [red: non-lesion, yellow: artifact, dark blue: ring pattern, and light blue: non-specific (potential malignancy/atypia)]. Segmented mosaic provided by Dr. Kivanc Kose, Dermatology Service, Department of Medicine, MSKCC. (b) Classification of fluorescence confocal microscopy videos of esophagus tissue as squamous tissue, Barrett’s esophagus, or dysplasia. Top: Barrett’s esophagus (left) and dysplasia (right) microscopy images with regions most relevant for training an automated classifier colored (red/orange) in a gradient-weighted class activation map. Bottom: confusion matrix for the trained deep-learning model. Reprinted from Ref. 129; CC BY 4.0. (c) 3D segmentation of the dermal–epidermal junction in RCM image stacks. Top image shows a segmentation of one *en face* plane (red: epidermis, yellow: uncertain transition region containing dermal–epidermal junction, blue: dermis). Bottom image shows epidermal lower boundary surface extracted with a segmentation algorithm. Reprinted from Ref. 130. (d) Automated segmentation of corneal nerve fibers for morphology assessment to quantitatively evaluate diabetic sensorimotor polyneuropathy using RCM. Images show extracted nerve fiber skeleton (left), detected intersection and end points (center), and final segmentation superimposed on original image (right). Receiver operating characteristic curves show the accuracy of four proposed machine-learning models in identifying nerve fibers. © 2017 IEEE; reprinted, with permission, from Ref. 131.

Another early application of AI approaches will be for improving the basic processes of image annotation (to guide human interpretation), rather than for definitive diagnosis. Examples in progress include automated segmentation of diagnostically relevant microstructures such as cellular patterns, nuclei, vasculature, or nerve fibers [Figs. 5(a) and 5(d)];^116,131^ automated detection of the dermal–epidermal junction in RCM stacks of melanocytic skin lesions to highlight the most diagnostically relevant depths [Fig. 5(c)];^130^^,^^132^ and detection of artifacts in RCM mosaics^133^^,^^134^ and probe-based confocal laser endomicroscopy images^135^ to assess image quality. Finally, progress is also being made in AI classification of IVM images to augment human diagnosis, such as detection of dysplasia/cancer in Barrett’s esophagus with accuracy approaching that of human observers [Fig. 5(b)].^129^

## 4 Summary and Outlook

**Fig. 6:**
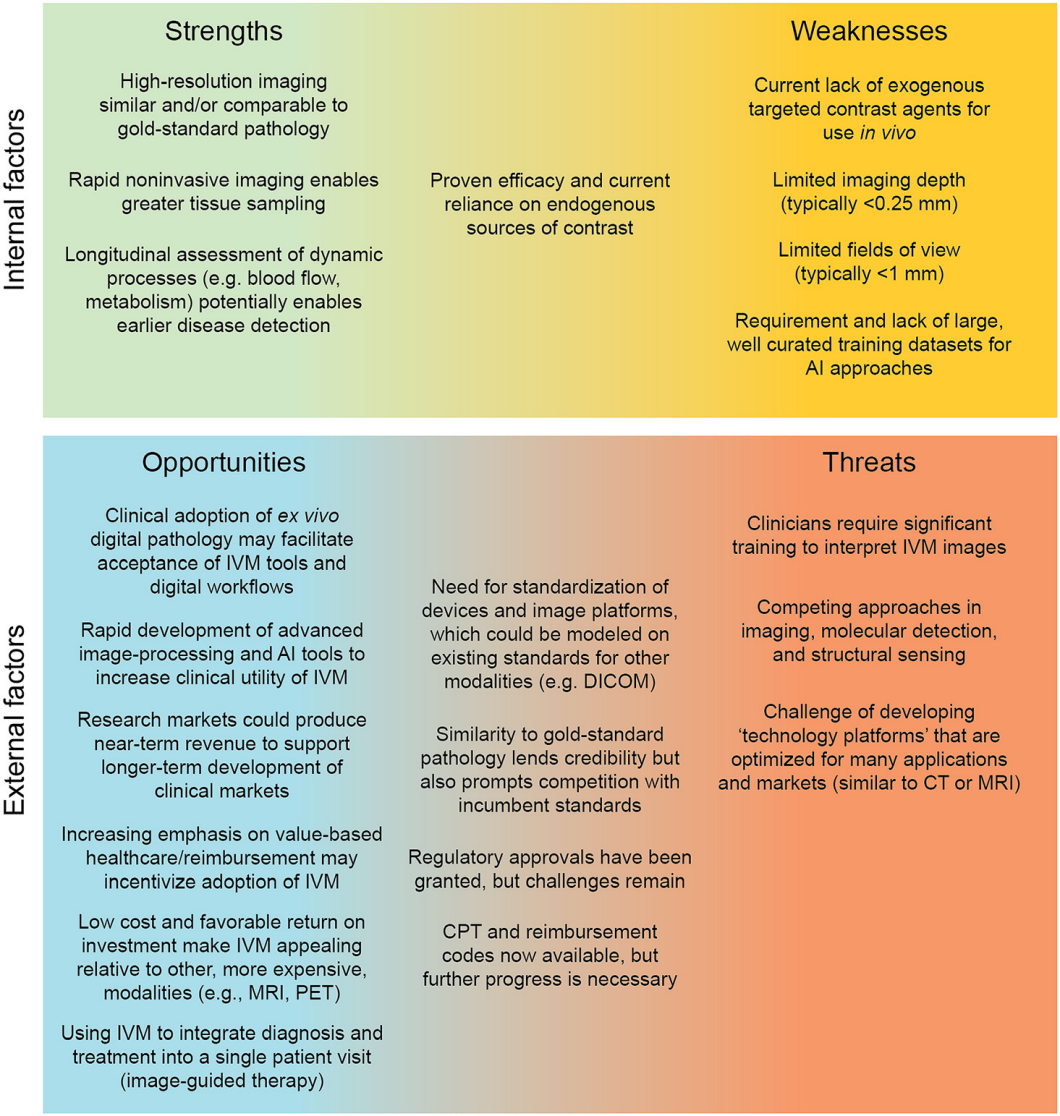
SWOT analysis for the clinical adoption of IVM.

In this perspective article, we have sought to provide a high-level overview of some of the key technical and translational considerations for the field of IVM. Our goal has been to provide a roadmap for others in the field to guide future progress. Figure 6 summarizes our key points in the form of a strengths, weaknesses, opportunities, and threats (SWOT) analysis.

In terms of the intrinsic strengths of IVM, a major advantage of IVM over other diagnostic platforms is that it provides imaging data that are similar and/or comparable to the established “gold standard” of anatomic histopathology, thus facilitating clinician interpretation and acceptance. The added benefit of doing so noninvasively, and with the ability to visualize dynamic processes longitudinally over extended time frames, provides substantial added value to IVM.

Early successes with endogenous “label-free” contrast, in particular RCM for dermatology applications, are encouraging, but also reveal fundamental limits with native non-specific contrast mechanisms and the value of exogenous targeted agents. The present lack of exogenous agents to complement endogenous contrast mechanisms represents a potential weakness from a clinical adoption standpoint. IVM, as a high-resolution optical method packaged into a portable device, is also fundamentally limited in terms of imaging depth and field of view, and therefore, will not be able to address all clinical needs. However, many researchers and industry groups are continuing to refine and validate new clinical applications. Ultimately, successful clinical adoption will require synergistic approaches that cleverly integrate new IVM approaches with existing standard-of-care methods and workflows.

A number of external factors have created opportunities in recent years to accelerate the clinical adoption of IVM technologies. Perhaps the largest opportunity is to leverage the recent FDA approval of digital pathology platforms, along with rapid research progress in AI methods for computer-assisted diagnosis and treatment guidance,^136^^–^^139^ to drive clinical acceptance of IVM. A large number of research efforts are also underway to develop targeted optical imaging agents for preclinical and clinical applications. The ability of IVM to serve these research and clinical markets may facilitate commercialization. In addition, increasing pressure on clinics in the USA and globally to deliver improved care while managing costs, combined with the relatively low cost of IVM, could provide an incentive for clinicians to adopt IVM tools. Low reimbursement rates for biopsy procedures have been a particularly challenging issue for clinicians within the USA for decades. For example, gastroenterologists are often reimbursed for only a limited number of endoscopic biopsies (or pooled sets of biopsies) with declining reimbursement rates, while reimbursements for benign biopsies are being eliminated altogether in certain dermatopathology settings.^140^^,^^141^ Finally, there are opportunities to use IVM within a one-stop shop patient-care paradigm in which diagnosis and treatment are integrated into a single patient visit.

Threats to IVM include the non-negligible learning curve for pathologists and proceduralists to learn how to interpret IVM datasets, which will differ from traditional histology images to some degree. Other noninvasive sensing and imaging techniques will also compete with IVM for clinical adoption, such as RF sensing and photoacoustic imaging. An additional challenge will be to develop technology platforms that can serve multiple large clinical markets, rather than niche applications, so that investors and medical institutions are more willing to pay for the commercial development and use of IVM tools. Multimodal approaches may be important to realize such technology platforms, in which case it will be essential to manage the relatively higher cost of such devices.

A number of external factors have the potential to have both a positive and negative influence on IVM. Existing imaging standards in radiology (e.g., DICOM and picture archiving and communication system [PACS]) could serve as a model for IVM, but could also complicate the standardization of these new technologies due to intrinsic differences between such modalities. As the field of *ex vivo* anatomic pathology continues to evolve and modernize, it will help to reinforce the value of tissue morphology in medical decision making. However, *ex vivo* pathology may also compete with IVM in certain clinical applications. Nevertheless, the intrinsic strengths of IVM (e.g., noninvasiveness, dynamic imaging, and real-time results) will continue to be highly attractive for a wealth of clinical scenarios, making IVM a valuable complementary technology to existing *ex vivo* pathology. Finally, recent successes in the regulatory approval and reimbursement of IVM tools in the USA, Europe, and elsewhere are encouraging for the field. With opportunities to leverage emerging AI technologies, novel contrast agents, and multimodal platforms to address a growing list of clinical applications, IVM remains an exciting and promising field and venture.
